# Abscess of the Brain

**Published:** 1934

**Authors:** E. Watson-Williams

**Affiliations:** Surgeon-in-charge, Ear, Nose and Throat Department, Bristol Royal Infirmary


### ABSCESS OF THE BRAIN,

### WITH REPORTS OF TWO CASES.

BY

E. Watson-Williams, M.C., Ch.M., F.R.C.S.,

*Surgeon-in-charge, Ear, Nose and Throat Department, Bristol Royal Infirmary.*

---

Abscess of the brain is a condition fortunately not very common, but none the less one very important to recognize. At the present time the mortality is high, and at least in part this is due to the fact that a large proportion of the cases are not recognized or are recognized only in the late stages, though even of those cases diagnosed and treated early a larger proportion die than should be the case.

### ÆTIOLOGY.

The initial infection may reach the brain in several ways.

*Trauma.*—As a result of trauma. (1) Direct, as in penetrating wounds ; (2) indirect, as with fracture of the base of the skull. We need not further consider these cases except to remark that (*a*) an abscess may arise months or years after the original injury, and (*b*) in some cases of abscess arising apparently by direct extension there is a history of trauma, such as a blow on the head.

*Hæmatogenous.*—By the blood-stream. (1) Arterial. (*a*) In acute pyæmia multiple abscesses in the brain may occur. (*b*) Arterial embolism may lead to *metastatic abscess*, usually single, where any chronic infective process is present, for example osteomyelitis, malignant endocarditis, even dental or tonsillar infection : the only important group of cases are those following lung suppuration, particularly bronchiectasis. (2) Venous septic thrombosis may lead to the extension into the brain of infective processes in the neighbourhood, and probably accounts for some of the abscesses that appear to have resulted from direct extension : the only other important group of this class is that in which the veins of the under surface of the frontal lobe carry infection via the cribriform plate and the ethmoidal vein from the angular vein, as may occur following a boil on the face or eyelid.

*Direct Extension.*—By direct extension from suppuration in the immediate vicinity, that is, usually, in the sinuses of the nose and particularly in the mastoid process. This last class is numerically by far the most important. The actual pathology appears to be: inflammation of the dura, inflammation of underlying arachnoid, matting together of dura, arachnoid and adjacent brain, extension of infection into the brain along the perivascular spaces. As has been said, in some of these cases the venous path is responsible ; and occasionally a metastatic abscess will arise from ear disease by the arterial route, in a part of the brain remote from the original focus.

The very great majority of abscesses secondary to sinus or aural disease arise in the course of chronic suppuration (as opposed to acute), and where the ear is concerned nearly always there is cholesteatoma formation. This may in part explain why brain abscess is uncommon in children, but not entirely why males are rather more often affected than females. The infecting organism is nearly always a streptococcus or a pneumococcus, in about equal ratio, but other organisms sometimes occur, and mixed infections are not uncommon.

### CLINICAL AND PATHOLOGICAL TYPES.

Once infection has occurred the course of the disease is governed by the relative virulence of the infection and the effectiveness of the response. Although no hard and fast line can be drawn, it is possible both clinically and pathologically to recognize three types.

*Suppurative Encephalitis.*—When the infection is of high virulence it produces a condition comparable rather to cellulitis than to abscess. In the centre of the affected area is a core of dead brain tissue ; round this a zone of engorged brain in which organisms and pus cells can be found, but with relatively little tissue reaction ; and outside this again a zone of œdema. Such a condition is sometimes described as "suppurative encephalitis" to emphasize the distinction from true abscess. Clinically these cases tend to have an abrupt onset, with headache, high fever, perhaps a rigor or convulsion, and to suggest the occurrence of meningitis. Localizing signs may be seen early, but are soon merged in the profound stupor from involvement of a large part of the brain in the œdema : although the results of operation are poor, a few recoveries are obtained.

*Sub-Acute Abscess.*—With an infection of lower virulence an actual abscess is formed, that is, a space filled with pus and deliquescent brain substance, the walls formed of necrotic brain tissue ; round this is a zone of active inflammation, which gradually forms a belt of granulation tissue; and outside this zones of engorged brain and œdema. In the course of time the granulation tissue organizes, and the abscess cavity, the size of which may increase very slowly or not at all, comes to be surrounded by a fibrous covering which is intimately joined to the surrounding brain, lined with a pyogenic membrane. This is the commonest variety of abscess, the clinical features of which are considered later.

"*Tumour.*"—At the extreme other end of the scale we find cases of very gradual evolution, where the clinical features are those of tumour formation rather than of abscess. Such a diagnosis having been made, the "tumour" should be removed complete in its capsule: when this is done, and the "tumour" cut across, it is found to consist of a thick mass of fibrous tissue, in the centre of which may be quite a small cavity containing pus, perhaps sterile.

### CLINICAL COURSE.

The clinical course of a "typical" brain abscess can be divided into three stages, although the division must be regarded as somewhat elastic, signs belonging to one sometimes appearing in another. The onset is heralded as a rule by signs of renewed activity in the adjacent suppurative focus; for example, an ear that has for years been painlessly discharging may become painful, while the discharge ceases.

*Invasion.*—The stage of invasion. In the majority of cases the onset is insidious. The patient complains of severe headache and of feeling very ill, but there may be nothing more to attract attention. The headache is usually "all over the head," but may sometimes show the special features described below. In children there may be no complaint of headache.

The temperature is somewhat raised, 100–101° F being common, and the pulse-rate is correspondingly increased. Occasionally there may be nausea, vague giddiness, or a feeling of "chill." In some cases, however, the onset is more dramatic, with a rigor and vomiting, or even convulsions—particularly with metastatic abscess. Or signs such as are described under the third stage (of compression) may be observed. Finally, a patient who has not previously complained may suddenly become comatose without warning.

*Latent Stage.*—The latent stage. Following the invasion, the patient may recover to a considerable extent, but not completely. The headache recurs, especially on lying down, or on any sudden change of position. The complexion takes on a dull, muddy tint. Appetite is poor or capricious, all food may taste unpleasant, and there may be considerable wasting—this is occasionally extreme. The tongue is furred and constipation may occur: nausea or even vomiting sometimes appear at this stage. The temperature is normal, as a rule, but shows some irregularity. If it is taken every half-hour, an occasional spike of high temperature, quite transient, may be observed. The pulse may show also a transient phase of slowing, 46 or 50 being counted for an hour or so only. The greatest changes, however, are mental, and often the relatives are the first to point out that "he isn't a bit like himself." The patient is irritable, cannot settle to anything like reading or writing and cannot concentrate; he may have periods when he is dull and lethargic; children are fretful, and *will not play with their toys; and periods may* occur when the changes of the next stage are seen. As a rule no useful information can be obtained by examining the eyes at this stage, but leucocytosis occurs and a lumbar puncture will show characteristic changes (p. 237). This stage may last four or five days or even several weeks, and merges gradually into the next.

*Compression.*—The stage of compression. This is the stage in which are seen the "classical" signs of brain abscess; but we should certainly not wait for it to develop if there is any possibility of making the diagnosis earlier. Although occasionally a brain abscess may reach a very large size, as a rule it is to the surrounding œdema that increase of intra-cranial pressure must be ascribed. The symptoms and signs are (*a*) those of cerebral compression and (*b*) localizing signs. *All* the signs and symptoms of this stage tend to vary in intensity from time to time, sometimes rather rapidly.

The mental state becomes still more obviously abnormal: the patient is drowsy, and may go off suddenly to sleep. He cerebrates slowly, and may even appear deaf (be sure that he is *not* deaf); on being asked a question several seconds or even minutes may elapse before he produces the answer, though the answer when it does come is correct. (In the mental changes seen in the worst cases of toxæmia from nasal sinusitis exactly the same symptom may appear.) Irritability, wasting, and the muddy complexion are still more marked. The temperature may be raised, but as a rule it tends to be subnormal, although irregular with an occasional rise, and the pulse to be slow—or at least slow in relation to the temperature. As pressure increases the pulse may show a somewhat rare, special character; that is, with inspiration it is rather slow, but with expiration it becomes very much slower for a few beats—for example, during inspiration 60, during expiration 44. Vomiting occurs, which is not specially related to food, and which sometimes shows a sudden "projectile" character. Headache is more severe and constant, though showing variations in intensity; it is often worse on the side of the lesion. After movement the patient may complain that "my head feels as if it is bursting," and this feeling may herald an attack of vomiting. Some degree of rigidity of the neck may be found, even when the abscess is supra-tentorial.

*Eye Signs.*—Photophobia may be noticed, or slight, fine nystagmus of labyrinthine type, if the eyes are turned well out to one side or the other—even though neither labyrinth nor cerebellum is affected: nystagmus due to one of these causes is discussed later (p. 242). Diplopia may occur, usually from paralysis of the external rectus; unfortunately this occurs in so many conditions that it is of little help in diagnosis, and it may not even indicate the side of the brain affected. Rarely diplopia is due to third-nerve paralysis, most often with cerebellar abscess, sometimes with a frontal lobe lesion, least frequently with a temporo-sphenoidal abscess. The pupil on the side of the lesion may be dilated, or may react more sluggishly than the other. In a proportion of cases papillœdema will be observed, particularly where the posterior fossa is involved: it tends to be more pronounced on the side of the lesion, but it is by no means constantly seen, even with an abscess of considerable size and duration. With severe papillœdema vision may be impaired, sometimes permanently.

*Lumbar Puncture.*—It cannot be too often emphasized that there is a definite risk in performing lumbar puncture when a brain (especially a cerebellar) abscess is present. In any case not more than 2 c.c.

should be withdrawn. The tests that give information of value are the cell-count, the differential cell-count, estimation of chlorides, and culture of the fluid; the estimation of glucose is of secondary importance in these cases, since the value of the examination is largely to distinguish early cases of abscess from meningitis, septic, meningococcal or tuberculous.

One may summarize the findings thus:—[1]

Pressure.—Normal 100—150 mm.=not more than two drops per second from the needle. Slightly increased in straining, stertor, meningismus, encephalitis, early septic meningitis or abscess; greatly increased in all other meningitis, tumour, late brain abscess.

Appearance.—Normal = water-clear. Clear or faint opalescence with brain (or extra-dural) abscess, early meningitis (any variety); turbid with established septic or meningococcal meningitis, or "leaking" brain abscess. 500 lymphocytes per c.mm. may produce only faintest opalescence, 200 polymorphs usually give definite turbidity.

Cells.—Normal 0—4 per c.mm. all mononuclears. With brain abscess *any* variation from this may be seen, the most usual being 10—30 cells per c.mm., of which 10—30 per cent. are polymorphs. If all mononuclears, probably tubercle, syphilis or tumour, encephalitis, poliomyelitis, etc. If polymorphs are 10—70 per cent. (10—500 per c.mm.) may be tubercle, syphilis, early septic meningitis or influenza. If polymorphs 70—90 per cent. (100—5,000 per c.mm.) leaking brain abscess, meningitis.

Bacteria.—Few in tubercle, meningococcal meningitis (at first). Streptococci, pneumococci mainly extra-cellular. If they grow on culture prognosis bad; turbid fluid with numerous dead bacteria and normal chlorides suggests leaking abscess.

Wassermann.—Positive reaction may occur with tumour or abscess apart from syphilis.

Glucose.—Normal 0·045—0·08 per cent.; slight *increase* with encephalitis, abscess; diminished with tuberculous or early septic meningitis; absent in septic or meningococcal meningitis or leaking abscess.

Chloride.—Normal 0·72—0·75 per cent., and with abscess; diminished in meningitis, typhoid, pneumonia; below 0·65 per cent.=tuberculous meningitis.

Other methods of investigation are skiagraphy, by which occasionally an abscess may be seen, ventricle puncture, or ventriculography (skiagraphy after injecting air into ventricles).

*Termination.*—If the condition persists unrelieved death will occur, either with deepening coma or respiratory paralysis from pressure, or from meningitis, or from rupture of the abscess into a ventricle (very high temperature, unconsciousness, stertor).

### LOCALIZING SIGNS.

The localizing signs of brain abscess are often few and transient. The abscess itself very rarely involves a part of the brain where lesions have characteristic signs; it is from involvement of surrounding parts in œdema (the extent of which may change rapidly) that signs occur. And therefore it is of the greatest importance to observe the patient closely and often in order not to miss these. The surest guide as a rule is the discovery of the original focus of infection; the abscess is seldom more than half an inch deep in the brain from this,* unless a metastatic abscess is present. Tenderness on percussion over the abscess is a valuable sign when observed: it is infrequent, and difficult to distinguish from tenderness due to the original focus.

*Frontal Lobe.*—Where the nasal sinuses are the starting-point the abscess is to be sought in the lower part of the frontal lobe: only when both sides show sinusitis will there be difficulty in deciding where to operate, and here there may be some help from the position of the headache. But frontal lobe abscesses are notoriously "silent," and may exist for long

* It may, of course, lie as deep as two inches from the external surface.

without even being suspected. One feature may give rise to suspicion—the patient is unduly optimistic, and although visibly getting worse is convinced that he is improving. Ocular palsies may sometimes help in the diagnosis.

*Temporo-sphenoidal Lobe.*—When the ear is to blame, even when we have decided which ear, we are no nearer knowing whether the abscess is in the temporo-sphenoidal lobe or in the cerebellum. The former is at least twice as common as the latter, but much more likely to be without localizing signs. These when they occur are most often due to œdema affecting the motor cortex above, or the fibres passing down from this through the internal capsule, with consequent *contra-lateral* weakness. Where the motor cortex is affected the lower part of the face is most likely to be affected, the corner of the mouth being weak (especially when at rest or on involuntary movement); the arm also may be affected, but the leg usually escapes. The reverse occurs if the motor tracts are involved at the internal capsule, the leg being most likely to suffer, the arm less so, while the face escapes. In the latter case the contra-lateral knee-jerk will be absent in the early stages; later it is brisker than normal, and the plantar reflex may be extensor, but the leg is "weak." Weakness may, of course, go on to paralysis.

When the left side is involved (extremely rarely the right) the "naming test" may be positive: the patient is shown a number of common objects and asked to name them one after another. If the test is positive he may name eight or ten correctly, and then find himself unable to name the next, although he knows quite well what the object is, and can pick out the right name from a list. Thus he may say:

"That's what you cut up your meat with; it's made of iron," etc., but he cannot remember that it is "a knife."

A sign rarely seen, but valuable when present, is a defect in the upper quadrant of the visual fields of both eyes on the side opposite the lesion, from involvement of the optic radiation as it sweeps back towards the calcarine fissure. Another rare sign is complaint of persistent bad smell (or taste), unrelated to food: this may indicate a lesion far forward in the temporo-sphenoidal lobe (or it may give the clue to unsuspected sinusitis).

*Cerebellum.*—When the cerebellum is involved the chances of complete absence of localizing signs are less. In the first place, nearly all cerebellar abscesses are secondary to lateral sinus thrombosis or to labyrinthitis—in about equal numbers—and lie in the region of the cerebellum nearest the structure concerned. Again, increase of intra-cranial pressure is earlier than with supra-tentorial abscesses, and so tend to be vomiting, bradycardia, neck rigidity and papillœdema. But the cerebellar cortex is "silent," and localizing signs depend on the deeper nuclei being affected. Signs are most likely to appear in the arm of the *same side*, and to a less extent in the leg; the face escapes. The limb in flaccid and weak and the tendon reflexes diminished; inco-ordination is present, and movements in which several joints are affected may be performed in stages, one joint being moved at a time; dysdiadochokinesis may be discovered. If the patient pulls against resistance, and the resistance is suddenly withdrawn, his hand flies away up, instead of being arrested in a short distance as normally. Nystagmus is present, and Rombergism, the patient tending to fall towards the side of the disease. Since several signs closely resemble those of labyrinthitis, it will be convenient to contrast the two conditions, remembering always that both may be present at once.

| *Cerebellar Abscess.* | *Labyrinthitis.* |
|---|---|
| Onset. — Gradual, symptoms increasing in intensity during several days. Vertigo may be mild. Vomiting often late, sudden, related to headache rather than food, perhaps without nausea. | Abrupt, with intense vertigo, vomiting, nystagmus. Symptoms tend to diminish in a day or two, but recur on movement. Vomiting related to movement, always with nausea. |
| Nystagmus.—Of cerebellar type, irregular, coarse, horizontal, quick component directed to side of disease, looking toward which makes nystagmus worse : on looking to healthy side slight fine nystagmus of " labyrinth " type may be seen. | Of labyrinthine type, fine, regular, mixed rotatory and horizontal, quick component directed to healthy side, looking toward which makes it worse ; lessened or abolished on looking to diseased side. |
| Falling.—Tends to fall toward the diseased SIDE, no matter what the position of the head. | Tends to fall in direction of diseased EAR. Thus, right ear diseased, head turned to right, tends to fall backward, head turned to left, falls forward. |
| Past-pointing.* — The arm of the *diseased side only* deviates outward. | *Both* arms tend to deviate toward side of diseased ear. |
| Ear. — If deaf, deafness of " EAR " type. Tuning-fork on mastoid heard better than on normal mastoid, on vertex heard better in diseased ear (if other ear healthy).<br>Syringing ear with hot water (110°) may bring on labyrinthine nystagmus to same side ; with cold water, to opposite side. | (Probably) complete (nerve) deafness to all sounds ; tuning-fork on vertex or mastoid heard only in opposite ear.<br>Syringing ear with either hot or cold water produces no effect at all. |

* The patient sits with eyes shut and arm extended forward, index finger touching fixed object ; raises hand above head, then brings it down in attempt to touch object again.

### DIAGNOSIS.

When all has been said, it must be admitted that the diagnosis of brain abscess in an early stage is fraught with difficulty, mainly because the symptoms are so little characteristic: "influenza" is the catch. Possibly the condition may be remembered if a patient without very definite signs complains of chilliness, nausea, or of feeling much more ill than one would expect, especially if he has chronic otitis media or bronchiectasis. When indefinite cerebral symptoms are present tuberculous meningitis or encephalitis may be suggested. To misinterpret cerebral signs in malignant endocarditis is perhaps of little practical significance. If the patient is comatose when first seen one has to consider uræmia, diabetes, alcohol and drugs: cerebral hæmorrhage in the posterior fossa may produce slight fever, pain and tenderness, rigidity of neck, nystagmus, (nerve) deafness and (permanent) changes in the labyrinthine reflexes. When there have been symptoms pointing to activity in old ear disease any intracranial complication is likely to be lateral sinus thrombosis, labyrinthitis, septic meningitis or brain abscess. A rigor in a subject of otitis media is nearly always evidence of thrombosis, a second may be considered conclusive (exclude malaria). Meningitis is of sudden onset, with very severe headache, and might lead to a mistake in diagnosis: it tends to get rapidly worse, and should be excluded at once, since it is one of the few surgical conditions where every hour makes a difference to the prognosis. Brain abscess is seldom so urgent as this, unless advice is sought only in the later stages, and then one may hope to observe at least some of the signs and symptoms described above; but a clear and complete picture is rare.

### TREATMENT.

There has been some discussion whether it is better to operate as soon as a diagnosis can be made with reasonable certainty, or to wait "until the abscess is walled off." My own views are definitely in favour of early operation: the risks the patient runs while waiting for a favourable issue are so much greater than those of exploration of the brain in proper hands. The details of operative technique we need not discuss.[2] The principles are: (1) large skin and muscle flap, wide resection of bone; (2) small opening in the dura, and careful and systematic exploration of the brain with a blunt trocar; (3) the abscess being found, fix a small drainage tube without removing the cannula; (4) apply a large dressing, and if the general condition is satisfactory do not touch the brain for at least a week—no probing, no irrigations, no "breaking down loculi." Recoveries have been recorded even after rupture of an abscess into a ventricle or cessation of respiration from medullary compression.

**Case 1.*** Abscess of left temporosphenoidal lobe secondary to otitis media; operation; recovery.

William B., aged 60. 24th June, 1934, complained of pain in left ear for fourteen days. The ear has run ever since he was a small boy, ceased six months back, discharged again five days ago, but without the relief of pain that he expected. Headache severe "all over." Definite rigidity of neck, moving head brings on pain in vertex, drowsy all the time. Pain and tenderness behind left ear, no redness or œdema. I saw him first on 25th, when rigidity of neck was barely noticeable. He was restless, throwing himself about and groaning with pain in left half of head; constantly nauseated, not vomiting—but he had vomited five times earlier in the day, and also on 23rd; no special character in vomiting. Temp. 96°; resp. 22. The pulse showed great variation in rate; with inspiration about 80, with expiration 48 or 50, but only for four to six beats. Average rate had been at 8.30 a.m. 84, at 9.30 a.m. 60, at 1 p.m.

88, at 2 p.m. as just described. He was rather confused, but understood simple questions, named objects correctly. Past-pointing could not be tested, vestibule not tested, no nystagmus, bone conduction increased. Knee-jerks absent, plantars flexor. Diagnosis: temporosphenoidal abscess.

Immediate operation: left radical mastoid operation. Bone cortex dense, acellular type, antrum full of mass of cholesteatoma which had "dissected away" all cancellous bone; no trace of ossicles found. Skull opened immediately above attic and antrum with nibbling forceps, brain explored through small dural incision with P. Watson-Williams's sphenoidal sinus cannula.[2] The first exploration reached an abscess 1 cm. above floor of middle fossa and 2 cm. from external surface of brain; about 15 c.c. of thick pus came out through cannula and afterwards through tube. When the dura was first opened the brain bulged tensely out, but pulsated well. A narrow drainage tube previously threaded over the cannula was slipped down to the tip without removing the cannula from the abscess, and secured by suture to the dura before the cannula was withdrawn: the pus grew streptococci on culture. The brain was dressed with strips of gauze soaked in glycerine, and the anterior part of the wound closed.

Next day (26th) very much better, takes fluids well, no headache, no vomit. 27th, pulse 88, temp. just above normal in evening, takes soft solids, quiet, intelligent (at least, his "normal"). 30th, whole ward stinks; outer dressing of sphagnum moss fibre, inner dressing not changed. 5th July, general condition very good; brain tube changed for first time (11th day); not irrigated. 3rd August, plastic closure of wound. Convalescence uneventful. 3rd September, he finds some difficulty in remembering *names* of things: left tinnitus, and some vertigo if he turns quickly. 18th October, very satisfactory; still occasional vertigo.

**Case 2.*** Abscess of right temporosphenoidal lobe secondary to otitis media; operation; recovery.

William G., aged 43. Came 5th July, 1934, complaining of severe frontal headache for one week, right aural discharge six days (he said not previously ever!), drowsiness and vomiting noted during a few hours. Temp. 100°, pulse 72, resp. 30. Seen 11 p.m. Pale, drowsy but quite sensible, prefers to lie

* These cases shown at the meeting of the Bristol Medico-Chirurgical Society, Dec. 12th, 1934.

in bed on left side, skin hot and dry, pulse full and regular, tongue furred, dry. Neck rigid but not true " retraction " ; groans with pain in the forehead, especially on any movement of head. Names objects correctly. Right ear: free purulent discharge, central perforation, no pain, tenderness or swelling ; bone conduction increased, Weber to right, no nystagmus or vertigo. Vomited several times earlier in the day apart from food, complete anorexia. Slight weakness of left corner of mouth. Knee-jerks, right weak, left normal ; plantars, flexor, right weak (? possibly =left reflexes +). Kernig's sign absent. No inco-ordination or ataxia (could not test walking). Lumbar puncture, pressure much increased, fluid cloudy; numerous polymorph. pus cells, no organisms seen (culture sterile), glucose absent. Diagnosis: cerebral abscess; 3 c.c. concentrated antistreptococcal serum intrathecally, 3 c.c. intramuscularly.

Immediate operation. Right radical mastoid. Cortex thick and dense, antrum full of cholesteatoma, pus offensive.

Dura of middle fossa had been exposed by erosion of bone above posterior part of antrum, and reddened there. Free exposure of dura by subtemporal route (as in Case 1) ;[2] dura pulsated well. Exploration of brain, abscess entered at first venture 2·5 c.m. from surface ; very offensive " watery-curdy " pus escaped cannula (culture later reported " streptocococci and leptothrix "). Drainage tube passed and secured, cannula removed, glycerine dressings.

6th July. General condition good, pulse 68. No headache, taking fluids well, no vomit. Optic discs normal. Later: pulse still slow (60—70), smell very offensive, sphagnum moss dressings.

13th July. Brain dressed and drainage tube changed for first time (8th day), no irrigation. Still gets headache on movement.

27th July. Up in chair ; no headache.

14th August. To convalescent home ; pulse now normal.

25th October, 1934. Very satisfactory recovery. No symptoms ; back at work.

REFERENCES.

[1] E. Watson-Williams, " Septic Meningitis," *Practitioner*, 1931, ii. 672.

[2] *Ib.*, *Emergency Surgery*, Hamilton Bailey, ii. 130. John Wright & Sons Ltd. 1931.

